# Child sex tourism – prevalence of and risk factors for its use in a German community sample

**DOI:** 10.1186/s12889-017-4270-3

**Published:** 2017-04-20

**Authors:** Thula Koops, Daniel Turner, Janina Neutze, Peer Briken

**Affiliations:** 1 Institute for Sex Research and Forensic Psychiatry, University Medical Center Hamburg-Eppendorf, Martinistraße 52, D-20246 Hamburg, Germany; 2 Department of Psychiatry and Psychotherapy, University Medical Center Mainz, Mainz, Germany; 3 Department of Forensic Psychiatry and Psychotherapy, University of Regensburg, Regensburg, Germany

**Keywords:** Child sex tourism, Child sexual abuse, Community sample, Prevalence, Risk factors

## Abstract

**Background:**

To investigate the prevalence of child sex tourism (CST) in a large German community sample, and to compare those who made use of CST with other child sexual abusers regarding established characteristics and risk factors for child sexual abuse.

**Methods:**

Adult German men were recruited through a German market research panel and questioned by means of an anonymous online survey. Group assignment was accomplished based on information on previous sexual contacts with children and previous use of CST. Characteristics and risk factors were compared between the groups using t- and Chi-square tests. Binary logistic regression analysis was performed to predict CST. Data collection was conducted in 2013, data analysis in January 2015.

**Results:**

Out of 8718 men, 36 (0.4%) reported CST use. The CST group differed from the nonCST group (*n* = 96; 1.1%) with regard to pedophilic sexual and antisocial behaviors as well as own experiences of sexual abuse. Social difficulties, pedophilic sexual interests, and hypersexuality were not distinct features in the CST group. Own experiences of sexual abuse, child prostitution use, and previous conviction for a violent offense predicted CST in a logistic regression model.

**Conclusions:**

This study is a first step to gain insight into the prevalence and characteristics of men using CST. Findings could help to augment prevention strategies against commercial forms of sexual abuse in developed as well as in developing countries by fostering the knowledge about the characteristics of perpetrators.

## Background

The commercial sexual exploitation of children represents a substantial global concern. According to the International Labour Organization (ILO) 1.8 million children were forced into prostitution and pornography in the year 2000 [1]. Other estimations add up to 10 million prostituted children worldwide [2]. It is estimated that more than US$5 billion are obtained through child prostitution every year [3]. Child sex tourism (CST), which is defined by the United Nations as “the exploitation of children for sexual purposes by people who travel locally or internationally to engage in sexual activities with children” (p.5) [4], accounts for a part of these earnings. While South-East Asia, Central America and Brazil are designated as countries with a long history of CST, with emerging destinations in South America, South, North-West, and East Africa, India and Mongolia, CST ‘users’ mostly originate from developed countries including European and North American countries, as well as Russia, Japan, Taiwan, Australia, and New Zealand [5].

Furthermore, CST is linked to the field of child and human trafficking [6, 7]. According to the United Nations human trafficking refers to the acquisition of people by force, fraud or deception for an improper purpose, for example forced labor or sexual exploitation [8]. The United Nations Children’s Fund (UNICEF) states that at least 1.2 million children are trafficked worldwide annually, primarily for the purpose of sexual exploitation [9]. A recent literature review on sex trafficking to the U.S. addresses its impact, causes, legal and economic consequences, and gives a brief description of the traffickers and victims [10]. According to the authors, human sex trafficking is not just related to a set of other crimes, such as fraud, extortion, coercion, or rape, but is in fact “the fastest growing form of international and intranational commerce and crime” (p. 155). The examination of the extent of trafficking – and consequently also of further research questions – is hindered by definitional and political aspects, as well as the illicit and hidden nature of the phenomenon. Schauer and Wheaton [10] emphasize both the necessity to research the trafficking of children as a special facet of human trafficking, as well as the serious lack of data concerning the “final consumers of sex trafficking” (p. 165). These information are needed to develop appropriate prevention strategies against commercial forms of sexual abuse in developed as well as in developing countries. Current prevention strategies against CST mainly address the education and sensitization of the general public and the training of tourism professionals [11], as well as countries’ legislation concerning CST, e.g. in terms of an elimination of the requirement of double criminality, according to which the crime would have to be illegal in both the home country and the location of the conduct [12]. According to the German Penal Code German citizens who commit sexual offences in another country may be legally charged for these offences, however, such an extraterritoriality component, allowing the prosecution of citizens for crimes committed outside of their home country, does not exist in every nation [12, 13]. In 2003 the US government passed the PROTECT Act that made it illegal for US residents to travel to foreign countries with the intention to have sex with minors [14]. Furthermore, the PROTECT Act included the request for the implementation of measures that lead to an early identification of possible abusers or of those who are at risk to become abusers when travelling to a foreign country [14].

It was suggested that CST might be motivated by the impression of anonymity arising from being in a foreign country [14] or prejudiced assumptions about cultural or “natural” differences in the sexual willingness of children in the respective destinations [15]. In this context, George and Panko [14] established a conceptual model of the “CST ecosystem” which states biological, psychological, and situational factors influencing the motivation for CST. They further distinguish between “elective” and “core sex tourists”, based on Klain’s [16] proposal that some individuals on leisure or business trips make unplanned use of CST when an opportunity is provided to them (“elective sex tourists”), whereas others’ precise purpose of the trip is sexual contact with a child (“core sex tourists”; [14]). Although much is known about the characteristics of the victims of CST, the systematic evaluation of the psychological and criminological features of CST perpetrators has been mostly neglected so far.

Previous research has identified various personality traits that can frequently be found in child sexual abusers and that increase the likelihood that someone will commit a sexual assault against children. It can be hypothesized that these characteristics are of relevance in CST perpetrators as well. Pedophilic sexual interests and antisocial behaviors have been described as the most important risk factors for child sexual abuse [17, 18]. However, own experiences of sexual abuse [19], hypersexuality [20] (for a critical discussion of the construct of hypersexuality in the context of child sexual abuse see [21]), and the use of child pornography [22] are further significant risk factors for child sexual abuse.

The present study aims at investigating the prevalence of CST in a large German community sample, and comparing those who made use of CST with other child sexual abusers from the community sample who did not engage in CST regarding the selected characteristics and risk factors.

## Methods

### Procedure

The present study was conducted in the context of a broader research project. Within an interdisciplinary network the project aimed at evaluating the frequency, causes, circumstances and negative effects of sexual assaults against children in different settings in order to improve current prevention and management approaches (for a detailed description see [23]). The present data were collected in 2013 by means of an anonymous online survey assessing the prevalence of pedophilic sexual interests and behaviors, as well as several related risk factors for child sexual abuse in men from the German community.

Participants were adult German males (≥18 years), who were contacted in consideration of population representativeness regarding age and educational level. All participants were recruited via a market research panel and were offered 20€ (approximately US$25) for completing the survey. Registration at the market research panel is voluntary and includes the request of demographic data. Men who fit the study’s inclusion criteria were contacted. They were informed about the aims and complete anonymity of the study.

The study was approved by the ethical review board of the German Psychological Society (DGPs).

### Participants

10,538 participants started to fill in the survey. Data of 1327 individuals (12.6%) were incomplete, another 493 (4.7%) withdrew their consent to the study and were excluded from data analysis. This resulted in a sample of 8718 male participants aged 18 to 89 years (M = 43.5, SD = 13.7). The present sample differed from the male German population with regard to age and educational level, manifesting an overrepresentation of people with higher education and aged between 30 and 49 years and an underrepresentation of people with lower education and over 65 years. Correlations between age categories, educational level, or their interaction and reported child pornography consumption as well as sexual behavior and fantasies involving children were ruled out (for underlying calculations and further descriptions see [24]). Study group assignment was based on reported previous child sexual abuse perpetration (see *Group Assignment in Measures section*) and previous use of CST. One-hundred-thirty-two participants (1.5% of the sample) admitted having sexually abused a child in the past. Among these, 36 individuals (27% of participants who disclosed a perpetrated child sexual abuse and 0.4% of the whole sample) had made use of CST. Of participants who did not disclose a perpetrated child sexual abuse (*n* = 8586), nearly 99% had passed school graduation, 30% of them after 10 years (comparable to a Middle School degree) and 56% after 13 years (comparable to a High School degree). Data on age are congruent with those of the whole sample (age range 18 to 89 years, M = 43.5, SD = 13.7). Participants with a history of child sexual abuse perpetration were slightly younger and less educated from a descriptive perspective. The two groups relevant to the research question are as follows:

- Group 1: participants who reported both perpetration of child sexual abuse and CST (CST; *n* = 36),
- Group 2: participants who reported perpetration of child sexual abuse and no CST (nonCST; *n* = 96).

Mean age and educational level of the groups are presented in Table 1. No significant differences between the two groups were found.

**Table 1:** *Age and educational level in child sex tourists (CST) and other child sexual abusers (nonCST* *)*

	CST (*n* = 36)	No. incl. *(n)*	nonCST (*n* = 96)	No. incl. *(n)*	-^b^/T	*p*	ϕ/d
Age *M (SD)*	39.80 (SD = 11.67)	35	41.26 (SD = 13.40)	94	.567	.572	.11
Educational level (years in school; *n (%* ^*a*^ *)*)							
- still in school	2 (5.6%)	36	0	94	-	.016	.30
- graduated after 9 years	11 (30.6%)	36	13 (13.8%)	94			
- graduated after 10 years	12 (33.3%)	36	33 (35.1%)	94			
- Graduated after 13 years	11 (30.6%)	36	47 (50.0%)	94			
- No graduation	0	36	1 (1.1%)	94			

*M* mean; *SD* standard deviation; *No. incl*.: number included

^a^Percentages refer to each number included *(n)* ^b^ Fisher’s Exact Test (expected frequency of 4 categories <5)

### Measures

#### Group assignment

Previous child sexual abuse was explored using a shortened 24-item version of the self-report Explicit Sexual Interest Questionnaire (ESIQ; [25]), whose reliability and validity have been demonstrated [26]. Internal consistency for the present sample was acceptable to good [24]. The questionnaire includes three items to evaluate sexual fantasies (“I find it erotic to see a …‘s body through the clothes”, “I get excited when I imagine that a … stimulates me”, “I find it erotic to imagine having sex with a …”) and sexual behaviors (“I have sexually caressed a …”, “I have tongue kissed a …”, “I have enjoyed getting my private parts touched by a …”) each item referring to men, women, prepubescent boys and girls (≤12 years). All items have a dichotomous response format (true/false) and relate to experiences one has had as an adult. Furthermore, participants were asked about child prostitution use (“Have you ever paid a child for sexual services?” [true/false]). All individuals who either affirmed child prostitution use or at least one sexual behavior involving a boy or girl were coded as child sexual abusers. The final assignment to Group 1 or 2 was based on the answers to the essential CST item (“Have you ever travelled to a foreign country in order to have sex with a child?” [true/false]).

#### Characteristics and risk factors

##### Sociodemographic characteristics

Participants were inquired about whether they had ever been or were in a romantic relationship with an adult that lasted longer than 2 years when they were adults themselves. Furthermore, one item assessed participants’ own abusive experiences (“Have you had sexual contact with an adult before the age of 14?” [true/false]).

##### Pedophilic sexual interest and behavior

Pedophilic sexual interest was measured via items about sexual fantasies involving boys and/or girls of the ESIQ (see above). Besides sexual behaviors with boys or girls assessed by the ESIQ, data were collected about whether or not participants had made use of child pornography (“Have you ever watched pornographic depictions of children, e.g., the nude genitals of children, to get sexually aroused after you were 18 years of age?” [true/false]) or child prostitution (see above).

Men who reported child sexual abuse were asked if they had committed the assault under the influence of alcohol. Additionally, they were asked how likely it was that they would sexually abuse a child under the age of 12 years again using a scale from 0 to 100. Participants were also asked if they had ever thought about seeking professional help because of their sexual interest in children.

##### Hypersexuality

Kafka [27] defines the threshold for hypersexuality to be seven or more orgasms per week. To assess the total number of orgasms the Sexual Outlet Inventory (SOI; [28]) was selected that measures how many orgasms participants had achieved during a regular week within the last month, no matter if through masturbation, intercourse, ‘wet dreams’, or otherwise.

##### Antisociality

Comparably to previous research a history of rule violations or previous convictions were taken as indicators for antisociality (e.g. [17]). Thus, participants had to indicate if they have ever been convicted of a property (e.g. theft, burglary), a violent (e.g. grievous bodily harm, battery) and/or a sexual offense (e.g. sexual assault, rape, child sexual abuse) in a dichotomous response format (true/false).

### Statistical analysis

Data of the CST and nonCST group concerning all risk factors were compared using two-sided t-tests for interval-scaled variables and chi-square (χ^2^) tests for categorical variables. When expected frequencies of certain categories lay below 5, Fisher’s exact test was applied [29]. Cohen’s d served as a measure of effect size for t-tests and the ϕ-coefficient for χ^2^-tests. Cohen [30] suggested to interpret d > .2 as a small, d > .5 as a medium, and d > .8 as a large effect. Regarding ϕ (the product-moment correlation between dichotomous variables coded with 0 and 1) he proposed .1, .3, and .5 as corresponding figures [31]. In order to avoid false significant results due to multiple testing (inflation of the alpha level; [32]) without reducing the tests’ power, sequential Bonferroni correction [33] was performed.

Controlling for outliers was required when examining the number of orgasms participants had achieved per week. For this purpose, the median absolute deviation (MAD; [34]) was computed. Since the analyzed subgroup of child sexual abusers is a marginal group in which psychological characteristics might not be normally distributed, a conservative threshold was chosen (three standard deviations; [35]). Consequently, only participants indicating orgasms ≤12 per week were included in the comparison. As some values were missing, the number of participants included in each comparison is designated in the tables.

Finally, all risk factors proved significant were comprised in a binary logistic regression analysis for the prediction of CST use. CST (yes/no) was entered as the dependent variable, while the risk factors were included as independent variables. All tests were performed using SPSS Statistics 21.

## Results

### Characteristics and risk factors

Results of the risk factor comparison between CST and nonCST are presented in Table 2. Participants in the CST group had themselves experienced sexual abuse significantly more often. Pedophilic sexual behaviors were reported to be significantly more common in CST users. More CST participants had consumed child pornography, had made use of child prostitution more often, and had shown more sexual behaviors involving boys. Use of child prostitution and sexual behavior involving boys obtained the largest effect sizes. Participants in the CST group estimated the risk of committing another sexual assault against a child as higher as participants in the nonCST group did. They also stated having thought about seeking professional help because of their sexual interest in children more often.

**Table 2:** *Group comparisons between child sex tourists (CST) and other child sexual abusers (nonCST) concerning risk factors*

	CST (*n* = 36)^1^	nonCST (*n* = 96)^1^			
	M (SD) / *n* (%)	M (SD) / *n* (%)	χ^2^/T	*p*	ϕ/d^c^
Sociodemographic characteristics					
No 2-year relationship	11 (30.6%)	26 (27.1%)	.16	.67	.03
Own abusive experience	26 (72.2%)	24 (25.0%)	24.81	< .001*	.43
Pedophilic sexual interests and behaviors					
Sexual fantasies involving boys: - dimensional *(M (SD))* - true/false *(n (%))*	.36 (SD = .42)	.27 (SD = .39)	−1.15	.25	.26
	18 (50.0%)	35 (36.5%)	-	-	-
Sexual fantasies involving girls: - dimensional *(M (SD))* - true/false *(n (%))*	.42(SD = .42)	.39 (SD = .41)	−.30	.77	.06
	21 (58.3%)	52 (54.2%)	-	-	-
Sexual fantasies involving boys or girls	22 (61.1%)	65 (67.7%)	.51	.48	.06
Sexual behaviors involving boys: - dimensional *(M (SD))* - true/false *(n (%))*	.43 (SD = .40)	.18(SD = .27)	−3.38	.001*	.78
	23 (63.9%)	37 (38.5%)	-	-	-
Sexual behaviors involving girls: - dimensional *(M (SD))* - true/false *(n (%))*	.44 (SD = .43)	.32 (SD = .26)	−1.57	.12	.38
	22 (61.1%)	69 (71.9%)	-	-	-
Child pornography	28 (77.8%)	33 (34.4%)	19.84	< .001*	.39
Child prostitution	28 (77.8%)	5 (5.2%)	73.54	< .001*	.75
Alcohol during sexual assault	13 (39.4%)	19 (21.3%)	4.05	.04	.18
Likelihood of future sexual assaults	48.00(SD = 35.33)	14.24 (SD = 22.99)	−5.03	< .001*	1.26
Thought about seeking professional help	14 (43.8%)	13 (14.9%)	11.07	.002*	.31
Hypersexuality					
Seven or more orgasms per week	2 (6.9%)	13 (15.5%)	-^b^	.35	.11
Antisociality					
Previous conviction for property offense	10 (31.3%)	9 (10.2%)	7.78	.01	.26
Previous conviction for violent offense	18 (56.3%)	6 (6.8%)	35.84	< .001*	.55
Previous conviction for sexual offense	12 (37.5%)	9 (10.2%)	12.09	.001*	.32

M: mean; SD: standard deviation

^1^Numbers of cases included vary slightly for some calculations

* Still significant after sequential Bonferroni correction

^a^Fisher’s Exact Test (expected frequency of 3 categories <5)^b^ Fisher’s Exact Test (expected frequency of 1 category <5)

^c^d > 0.2: small effect, d > 0.5: medium effect, d > 0.8: large effect

ϕ > 0.1: small effect, ϕ > 0.3: medium effect, ϕ > 0.5: large effect

Participants in the CST group had been convicted more often for a violent or sexual offense, demonstrating a large and a medium effect.

### Prediction of CST

A prediction of CST use was accomplished containing all risk factors that had shown to be distinctive. Results of the regression analysis are presented in Table 3.

**Table 3:** *Binary logistic regression analysis predicting CST*

Predictors	*B*	*SE B*	*e* ^B^	95% CI for *e* ^B^	
				*Lower limit*	*Upper limit*
Own abusive experience	2.33*	1.03	10.29	1.37	77.29
Sexual behaviors involving boys	2.97	1.76	19.62	.63	613.38
Child pornography	−1.33	1.21	.27	.03	2.83
Child prostitution	5.60***	1.38	270.67	18.25	4015.45
Likelihood of future sexual assaults	−.01	.02	1.00	.96	1.03
Thought about seeking professional help	1.41	1.21	4.1	.38	44.27
Previous conviction for violent offense	4.59**	1.47	98.62	5.49	1772.76
Previous conviction for sexual offense	−2.46	1.54	.09	.004	1.74

R^2^ = .55 (Cox & Snell), .80 (Nagelkerke). Model χ^2^(8) = 95.40, *p* < .001. B: regression coefficient; SE B: standard error; *e* ^B^: exponentiated *B*; CI: confidence interval; *: *p* < .05; **: *p* < .01; *** *p* < .001

Only *B*-coefficients for own experience of sexual abuse, use of child prostitution, and previous conviction for a violent offense reached statistical significance. Especially the use of child prostitution and a previous conviction for a violent offense proved to have a comparatively high predictive value for use of CST.

## Discussion

In this study 1.5% (*n* = 132) of German men who participated in the survey reported having at least once in their life engaged in a sexual contact with a child. This percentage is in line with previous findings [36–38]. Thirty-six participants (0.4%) indicated CST activities in the past. In general, estimating the prevalence of men who have abused a child within community samples is difficult, because most data only include those who got into conflict with the legal system and thus do not capture undetected sexual assaults [39]. For this reason, comparative information on child sex tourists is even rarer. Bernard [40] found 51% out of 73 pedophilic activists (members of the Working Group of Pedophilia) to have travelled abroad for sexual purposes, but evidently, those figures are not suitable for generalization, as the sample probably consisted of men with particular sexual interests. Therefore, the results of this study might give a first orientation about the prevalence of CST use among German men that could be representative for a modern western country.

The manifestation of risk factors diverged in some respects between users of CST and other child sexual abusers. The core features standing out in the CST group were own experiences of sexual abuse, pedophilic sexual behaviors, and antisocial behaviors. Interestingly, the two groups exhibited no differences concerning pedophilic sexual interests and hypersexuality. This is important because previous research has shown that pedophilic sexual interests and sexual preoccupation (for a definition of the term see [41]) are two of the most important risk factor for child sexual abuse [17]. These findings suggest that a higher degree of antisocial behaviors might be a prerequisite for engaging in CST. It might be possible that travelling to another country to have sexual contact with children presents a considerable obstacle even for child sexual abusers and thus more antisocial tendencies are needed to overcome this hurdle. The same accounts for the higher rate of sexual behaviors with children in the CST group because antisociality is also associated with contact sexual offending. A study by Lee et al. [42] demonstrated higher levels of antisociality in exclusive child molesters compared to exclusive child pornography consumers.

In terms of future prevention strategies it is important that - despite antisocial characteristics - CST users reported a higher perceived likelihood of another sexual assault in the future but also more often thoughts about seeking professional help. One possible interpretation could be that CST users perceived lower inhibition towards future sexual assaults against children due to more general and sexual self-regulation problems, which would also be in line with the higher rate of antisocial behaviors observed in this group. Furthermore, they might feel more distressed by the lack of these skills. However, it is also possible that CST users had thought more often about professional help because they had been convicted more often for their offenses or because they were already under mandated treatment. Nevertheless, this issue needs to be examined in more detail in future studies because it could be an important goal for prevention programs addressing men with a risk to use CST since they seem to be more willing to engage in treatment.

Own abusive experiences among child sexual abusers are well documented [19, 36, 43, 44]. This characteristic seems to be specifically marked in users of CST. Of course most victims of child sexual abuse do not become sexual offenders, and other studies failed to find a connection between child sexual abuse and victimization [45]. This aspect therefore needs to be treated with caution.

The regression analysis confirmed the influence of own experiences of sexual abuse, child prostitution use, and previous conviction for a violent offense as predictors of CST. Although the present data reflect the importance of the mentioned factors, some variance still remaining unexplained signifies that other relevant risk factors need to be detected that lead men to use CST. Moreover, additional information about the circumstances in which CST users take advantage of child prostitution apart from CST should be collected.

This study has several limitations. First of all, the groups in the focus of statistical analysis were rather small, which is why the degree to which these results can be generalized to the population is restricted. Results of regression analysis might also be affected by the sample size. But as the use of CST is probably and hopefully a relatively rare phenomenon and the examined sample is large, the new insight still is remarkable. Further, the information gathered only draws upon self-report data. While self-report is not necessarily reliable, especially not when it concerns criminal acts, there is hardly an alternative to anonymously assess such confidential data in a community sample. CST as well as own experiences of sexual abuse were also only assessed using one dichotomous item each, whereby the various forms of sexual violence are not taken into account. However, given the breadth of subject matter covered in the survey, such level of detail was not possible. Due to item wording, it cannot be ruled out that some participants travelled to another country for CST purposes without putting this into action. It is also possible that some of those having sexually abused a child either refused to participate, particularly after being informed about the study purpose, or withdrew their consent after completing the survey, whereby results might be biased. Furthermore, social desirability might play a role when answering the questions, given that child sexual abuse and pedophilia are associated with a strong stigma in our society [46]. With regard to both antisociality and hypersexuality, it needs to be considered that behaviors only partially serve as indicators.

Further research should explore CST users in a more detailed manner to foster the knowledge about their character and life circumstances. Subsequent studies should focus more concretely on specific aspects of CST (destinations, strategies to approach children, kinds of sexual contact) and on psychological factors as well (e.g., cognitive distortions, self-regulation) and the sexual offenses they committed in their home country. Comprising different sources of information in addition to self-report data might be useful.

## Conclusions

The present study to our knowledge is the first that assessed the prevalence and different risk factors for CST in a large community sample. A first step was made to gain insight into the characteristics of these men. As stated in the Convention of the Rights of the Child [47], countries are supposed to create appropriate prevention strategies against the “exploitative use of children in prostitution or other unlawful sexual practices” (Art.34). The World Health Organization (WHO) has augmented its prevention activities against child maltreatment by assisting with the implementation of such strategies and providing guidelines [48]. Nonetheless, the organization points out that epidemiological studies concerning the prevalence of child maltreatment are required to emphasize the topic’s seriousness [49]. This study was conducted with compliance to this goal. There is evidence that CST users form a particular and maybe high-risk group among child sexual abusers that could be reached by suitable prevention strategies.

## Abbreviation

CST: Child sex tourism
